# Self-Responsive Fluorescence Aptasensor for Lactoferrin Determination in Dairy Products

**DOI:** 10.3390/molecules29133013

**Published:** 2024-06-25

**Authors:** Hao Liu, Xibao Gao, Hongwei Qin, Mengmeng Yan, Chao Zhu, Linsen Li, Feng Qu

**Affiliations:** 1 Department of Physical and Chemical Inspection, School of Public Health, Cheeloo College of Medicine, Shandong University, Jinan 250000, China; liuhao981202@163.com; 2 Institute of Quality Standard and Testing Technology for Agro-Products, Shandong Academy of Agricultural Sciences, Jinan 250100, China; qhw01@163.com (H.Q.); ynky202@163.com (M.Y.); 3 Shandong Provincial Key Laboratory Test Technology on Food Quality and Safety, Jinan 250100, China; 4 Key Laboratory of Molecular Medicine and Biotherapy, School of Life Science, Beijing Institute of Technology, 5 South Zhongguancun Street, Beijing 100081, China; forrestlee2017@foxmail.com (L.L.); qufengqu@bit.edu.cn (F.Q.)

**Keywords:** aptamer, self-responsive, fluorescence aptasensor, lactoferrin detection

## Abstract

In this study, a self-responsive fluorescence aptasensor was established for the determination of lactoferrin (Lf) in dairy products. Herein, the aptamer itself functions as both a recognition element that specifically binds to Lf and a fluorescent signal reporter in conjunction with fluorescent moiety. In the presence of Lf, the aptamer preferentially binds to Lf due to its specific and high-affinity recognition by folding into a self-assembled and three-dimensional spatial structure. Meanwhile, its reduced spatial distance in the aptamer–Lf complex induces a FRET phenomenon based on the quenching of 6-FAM by amino acids in the Lf protein, resulting in a turn-off of the fluorescence of the system. As a result, the Lf concentration can be determined straightforwardly corresponding to the change in the self-responsive fluorescence signal. Under the optimized conditions, good linearities (R^2^ > 0.99) were achieved in an Lf concentration range of 2~10 μg/mL for both standard solutions and the spiked matrix, as well as with the desirable detection limits of 0.68 μg/mL and 0.46 μg/mL, respectively. Moreover, the fluorescence aptasensor exhibited reliable recoveries (89.5–104.3%) in terms of detecting Lf in three commercial samples, which is comparable to the accuracy of the HPCE method. The fluorescence aptasensor offers a user-friendly, cost-efficient, and promising sensor platform for point-of-need detection.

## 1. Introduction

Lactoferrin (Lf), as a non-heme iron-binding glycoprotein, is one of the most representative biologically active proteins widely present in mammalian milk [1]. It was first extracted from milk in 1939 [2] and was later identified as Lf with a molecular weight of approximately 80 kDa [3]. Beyond its ability to regulate iron metabolism, Lf has the potential to enhance the body’s antibacterial, antiviral, intestinal flora, and immunity-regulating capabilities, etc. [4]. In particular, these efficacies make Lf a functional ingredient used by infant formula manufacturers to complement their formulas and make them breastfeeding-friendly for infants and young children [5]. As a result, Lf-containing infant formulas are highly sought after by consumers, especially for non-breastfeeding families [6]. In recent years, some countries have stipulated regulations on the maximum allowable addition of Lf into dairy products, such as the US FDA and China with 100 mg/100 g, and the EU with 30–770 mg/100 g. However, due to its relatively high cost, to deceive consumers some unscrupulous traders produce infant formulas that contain much lower amounts of Lf than those labeled. Therefore, the development of Lf detection is of great significance for the quality control and efficacy assurance of infant formulas.

Currently, Lf detection in dairy products can be classified into three main categories, including chromatography, biosensors, and immunoassays [7]. Among chromatography, high-performance liquid chromatography (HPLC) enables a reliable distinction between different forms of Lf and has been intensively and extensively investigated and implemented [8], as well as the cutting-edge approaches such as monolithic cation exchange HPLC [9], HiTrap^TM^ Heparin HP column into HPLC [10], micro-batch resin extraction HPLC [11]. Recently, Yang et al. integrated amino acid-based isotope dilution LC–mass spectrometry into a mass balance approach to achieve SI-traceable purity assessment of bovine Lf with a low level of uncertainty (<2%) [12]. Apart from HPLC, some ion-exchange chromatography (e.g., pilot-scale monolithic IEC [13]) and high-performance capillary electrophoresis (HPCE) [14] are also attracting a lot of attention. Although chromatographic-based methods are typically traditional and effective methodologies, their application can be negatively affected by some aspects that involve inconvenient portability, the requirement for expensive instruments, and professional operators. With the rapid development of biometric components, biosensor-based detection is evolving rapidly, benefiting from its advantages of being simple, fast, and sensitive, including examples such as electrochemical biosensors [15] and molecularly imprinted polymer (MIP) biosensors [16]. For example, Judith et al. developed a vinylpirydin-based MIP biosensor with a selectivity coefficient of 9.91 that was nearly ten-fold greater than that without MIP. However, the biosensor methods still contained some shortcomings such as more cumbersome preparation and limited real-world applications. Radio immunodiffusion (RID) is simple in terms of operation and equipment and provides considerable sensitivity using kits, but its limitations are low throughput and poor precision [17]. Additionally, antibody-based immunoassays have been extensively incorporated into convenient products, e.g., the enzyme-linked immunosorbent assay (ELISA) [18], microfluidic papers [19], and lateral strips [20], while the discovery of antibodies generally involves long cycles of animal immunization experiments, as well as stringent conditions to guarantee their stability [21]. In view of the diversity of Lf functions and the current detection dilemma, it is highly essential to develop convenient, user-friendly, and effective methods for Lf detection.

Recent work by Qu’s group using CE-SELEX to gain aptamers against Lf with equilibrium dissociation constants (K_D_) of 20.74 nM has laid the aptasensor-related groundwork for Lf detection [22]. Aptamers are short strands of ssDNA or RNA, generally 15–90 nt in length with secondary structures such as hairpins and pseudoknots, and molecular weights of approximately 5–15 kDa, selected via the systematic evolution of ligands by exponential enrichment (SELEX) [23,24]. Aptamers are functionally similar to antibodies with high specificity and affinity, which has led to the term “chemical antibody” [25]. They are easy to produce through in vitro chemical synthesis and therefore have little variation between batches, are relatively inexpensive, are usually supplied as stable powders, can be stored for long periods at temperatures of 4 °C or even RT, and are therefore more readily available than antibodies stored at ultra-low temperatures [26,27]. In addition, aptamers have a fairly small molecular size, low immunogenicity, easy cell entry, and high bioavailability, and thus have potential applications in aptamer drug discovery, disease diagnosis, and therapy [28,29]. Furthermore, aptamers can be chemically modified, structurally chimeric, open to signal enhancement and amplification, enzyme-catalyzed, programmed, and combined with advanced materials to create multifunctional and versatile aptasensors for environmental detection, food safety, and disease diagnosis [30,31]. Among them, fluorescence-based aptasensors are the most frequently applied approaches because of their high responsiveness, great sensitivity, and general availability [32]. Depending on how the fluorescence signal is generated, the corresponding sensors can be classified into unlabeled and labeled types [33]. The unlabeled type usually uses dyes with some toxicity such as SYBR Green and PicoGreen as signal labels [34,35]. The labeled type often involves complex probe preparation, stringent enzyme involvement, and synthesis of nanomaterials, which increases the sensitivity and at the same time increases the difficulty of practical operation, limiting their practical applications [36]. Therefore, there is still room for improvement in the simplicity required for the convenient determination of Lf.

In this study, a self-responsive and user-friendly fluorescence aptasensor was developed for the sensitive detection of Lf in dairy products in view of the fact that amino acid groups in proteins have demonstrated the quenching of fluorescent groups by mechanisms [37,38,39] such as Förster resonance energy transfer (FRET) [39]. Herein, 6-FAM-labeled aptamers were employed as specific recognition elements and fluorescent reporter probes, Lf as target proteins and their amino acids as quenching acceptors. In the presence of Lf, the aptamer was specifically targeted for recognition of Lf by self-assembling and folding into a three-dimensional spatial structure. Meanwhile, its binding caused a reduced spatial distance that resulted in a quenching phenomenon through FRET on the energy donor–acceptor pair of the FAM moiety and amino acids, inducing a “turn-off” system. According to the reflected fluorescence signals, the corresponding Lf concentrations were directly determined with a reliable detection limit, and good recoveries, as well as the real application. The aptasensor has achieved a highly sensitive and handy detection approach through simple “mixing-and-detecting” operations, addressing the requirement to create uncomplicated and practical aptasensors.

## 2. Results and Discussion

### 2.1. Working Principle and Feasibility

**Figure 1:**
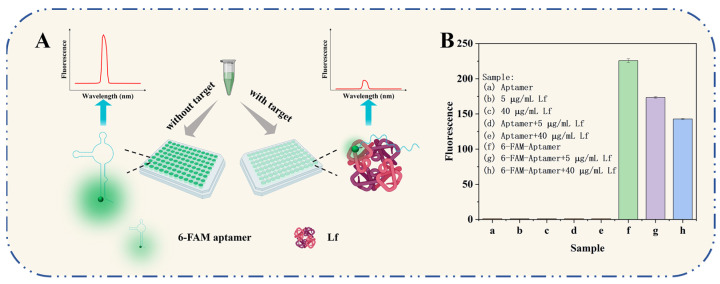
(**A**) The working principle of the self-responsive aptasensor for Lf detection. (**B**) Feasibility verification of the aptasensor.

The working principle of the self-responsive aptasensor is illustrated in Figure 1A. The sensor system consisted of only two components: aptamers and Lf targets, with the aptamer acting both as a specific recognition element targeting the Lf protein and as a fluorescence signaling reporter incorporated with fluorophores. In the absence of Lf, the combined superimposed effects of complementary base pairing and weak non-covalent interactions between molecules result in a specific and flexible spatial structure of the free aptamer oligonucleotide sequence. In this case, the fluorescent moiety of a 6-FAM-labeled aptamer gives off its own intense fluorescence. In the presence of Lf, the aptamer is recognized by Lf to form an aptamer–Lf complex by self-assembling and folding into a three-dimensional and inflexible spatial structure. In consequence, a phenomenon of a significantly reduced fluorescence of the aptamer was observed, which is speculated to be one possible explanation of Förster resonance energy transfer (FRET): the binding caused a reduced spatial distance that resulted in a FRET phenomenon based on the quenching of the aptamer by the amino acids in the Lf protein. The decrease in the fluorescence signal was associated negatively to the concentration of Lf in the samples. The presented aptasensor was carried out using a handy “mix-and-detect” operation undergoing a sequential reaction of self-assembly and self-response events, ensuring full coverage of Lf binding for robust determination and reducing accessibility to non-specific surface binding sites.

The sensing system’s feasibility is demonstrated in Figure 1B. The 6-FAM-labeled aptamer exhibited a unique enhancement in fluorescence, while the bare aptamer produced substantially no fluorescence. The mixture of 6-FAM-labeled aptamer and the addition Lf (5 μg/mL, 40 μg/mL) presented a decreased fluorescence, indicating that the aptamer would be more likely to specifically bind to its target Lf and more FAM moieties were quenched. The possibility could also be interpreted in terms of having previously developed a molecular docking mechanism for aptamer/Lf identification, which involves 18 amino acids among the Lf and 17 key bases among the aptamer and their binding collapsed into a monolithic, more rigid spatial structure, as well as, in turn, inducing FRET. The result provided preliminary evidence of the feasibility of the experimental principle.

### 2.2. Optimization of Experimental Conditions

**Figure 2:**
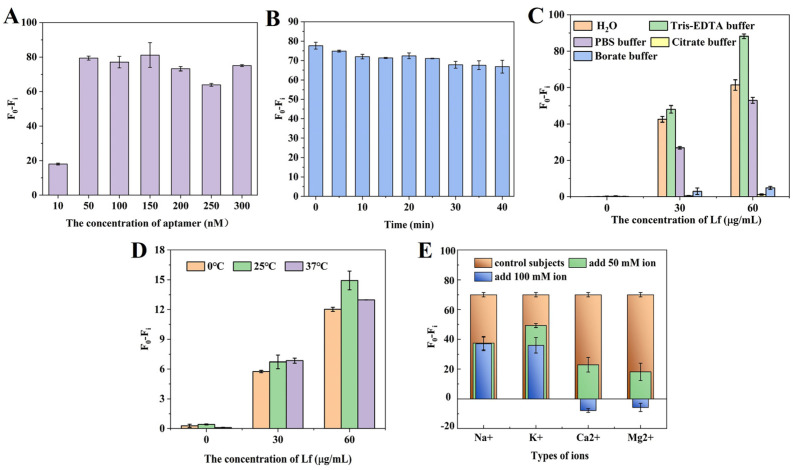
The optimizations of (**A**) the concentrations of aptamer at 10, 50, 100, 150, 200, 250, and 300 nM; (**B**) the incubation time between aptamer and Lf at five-minute intervals for 40 min; (**C**) the buffer solution by Tris–EDTA, PBS, H_2_O, citrate buffer, and boric acid buffer; (**D**) the reaction temperatures at 0, 25, and 37 °C; and (**E**) the ionic species (Na^+^, K^+^, Ca^2+^, Mg^2+^) and their strength at 50 mM and 100 mM.

Several parameters were carefully optimized to ensure the fast and accurate determination of Lf. Figure 2A shows the effect of different concentrations of aptamer on the fluorescence signal. The results revealed that aptamer concentrations from 50 nM to 300 nM had little effect on the fluorescence difference. The fluorescence difference is defined as (F_0_ − F_i_), where F_0_ is the fluorescence value of the aptamer in the absence of Lf and F_i_ is the fluorescence value of different concentrations of the aptamer after the addition of Lf. To ensure a suitable signal-to-noise ratio, an aptamer concentration of 50 nM was selected to participate in the latter reaction. Then, the incubation time of the aptamer with Lf was optimized. Figure 2B shows that the fluorescence values gradually increased with a longer time, but there was no significant difference within 40 min, which suggests that Lf was able to quickly open the specific spatial structure of the aptamer, and the specific binding of the two was very fast. For subsequent experiments, an incubation time of 10 min was therefore chosen. Figure 2C reveals that the buffers with different components or pHs presented significant distinctions, especially poorly in citrate buffer and borate buffer, which may be because these buffers contributed to structural stability differences or the conformation diversity of the aptamer and the Lf–aptamer complex. In these five buffers, the fluorescence difference was always greatest under the Tris–EDTA buffer regardless of the concentration of the aptamer, which may be related to the ability of this EDTA to stabilize the buffer environment by maintaining the ionic strength and pH that is essential for ensuring the folded conformation of the aptamer binds to the target. Therefore, the Tris–EDTA buffer was used for further experiments. Figure 2D illustrats the effect of different temperatures on the fluorescence intensity. At the same concentration, the (F_0_ − F_i_) values changed slightly with the reaction temperature. However, there was no significant trend. Therefore, in this sensor system, the effect of reaction temperature on fluorescence intensity was small, which indicates that the 6–FAM fluorescent dye was more stable at these three temperatures. For convenience, room temperature (25 °C) was used for further experiments.

Furthermore, the effect of ionic species and strength on the sensing system was investigated. In aptasensors, the ability of the aptamer to bind to the target depends on the molecular conformation of the aptamer, which is strongly influenced by various cations [40]. In some binding reactions, the presence of cations reduces the affinity between the aptamer and the protein, which is detrimental to their binding, and the effect of divalent ions is stronger than that of monovalent ions [41]. In some reactions, a low concentration of ions favors binding, while a high concentration of ions is detrimental to binding [42]. Figure 2E reveals that, at the same concentration, the effect of divalent ions on Lf was stronger than that of monovalent ions, which might be related to the fact that divalent ions have greater electrostatic interactions and higher hydration energies and that the divalent ions would lead to conformational rearrangement of the targeted aptamer in the buffer, lowering the binding rate of the aptamer to the target, and thus enhancing its fluorescence. Secondly, the binding of the aptamer to the target decreases with the addition of ions, which may be due to the shielding of the negative charge and the conformational change in the binding site of the aptamer. After the addition of ions, the cation interacts with the negatively charged phosphate group on the aptamer to form a binary or even ternary complex, resulting in a conformational change in the aptamer and a decrease in binding to the target. Therefore, Tris–EDTA buffer solution without added ions was used for subsequent experiments.

### 2.3. Sensitivity, Specificity, and Stability

**Figure 3:**
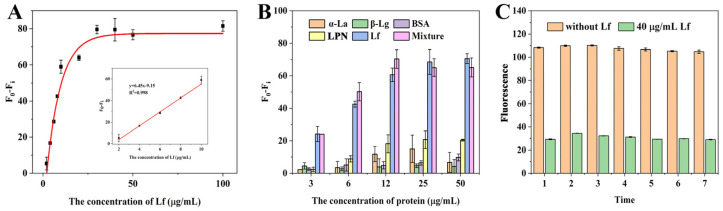
(**A**) The linear relationship between (F_0_ − F_i_) and the concentrations of Lf. (**B**) The specificity of the aptasensor strategy. (**C**) Stability assessment of the fluorescence aptasensors.

Under the optimization conditions, the sensitivity of the sensing system was evaluated with a standard Lf solution at various concentrations ranging from 2 to 100 μg/mL. As can be observed in Figure 3A, the fluorescence difference gradually increased with the increase in Lf concentration and reached a steady state when the concentration exceeded 30 μg/mL. A good calibration curve with a reliable correlation coefficient (R^2^) of 0.998 was fitted in the range of 2–10 μg/mL (Figure 3A inset) based on the linear relationship between the fluorescence difference (F_0_ − F_i_, vertical coordinate) and the Lf concentration (horizontal coordinate). The error bars are based on three measurements made in parallel at different concentrations. Meanwhile, the regression equation was derived as y = 6.45x − 9.15, along with a limit of detection (LOD) of 0.68 μg/mL (8.18 nM) based on the 3σ (σ = α/k, α = 1.4611 is the standard deviation of the blank signal, k = 6.45 is the slope of the standard curve). Thus, it could be concluded that the self-assembled and self-responding fluorescence aptasensor was capable of the quantitative determination of Lf. Although the LOD is moderate compared to other reported methods (Table 1), it is fully in accordance with the maximum allowable use such as in the US, EU, and China, together with a convenient “mixing-and-detecting” operation. Furthermore, these ELISA methods in Table 1 are based on the immune reaction between Lf and its antibodies, which are expensive reagents and complex preparations.

To verify the specificity of the aptasensor, the major proteins including α-lactalbumin (α-La), β-lactoglobulin (β-Lg), bovine serum albumin (BSA), lactopontin (LPN), and their mixture, were simultaneously evaluated to avoid the occurrence of false positives. As shown in Figure 3B, a similar and significant reduction in the fluorescence intensity of the aptasensor was observed in the wells containing Lf alone and the mixture (containing Lf, α-La, β-Lg, BSA, and LPN). In contrast, even with increasing concentrations, the other proteins showed almost no significant decrease, demonstrating that the self-responsive aptasensor showed good specific detection and determination of Lf. However, it can be observed that the proteins, especially α-La and LPN, still slightly affected the fluorescence signal above 12 μg/mL, suggesting that sample matrix effects should be considered in practical applications.

To evaluate the stability of the aptasensor, two sets of experiments were performed, including intra- and inter-day assays within 7 days. The fluorescence was measured using the Lf standard of 40 μg/mL. The coefficient of variation (CV) provides a relative measure of variability and has often been employed as an important guide to repeatability; higher CV values mean greater detection error and lower CV values mean more stable results. Figure 3C showed that there was no significant change in the fluorescence intensity of the aptasensors during the intra- and inter-day periods, and the calculated average CVs were very low, 0.004 and 0.008, respectively, which indicates that the aptasensors have good stability and reproducibility, as well as being ready for long-term usage.

### 2.4. Calibration Curve and Sensitivity in Spiked Matrix

**Figure 4:**
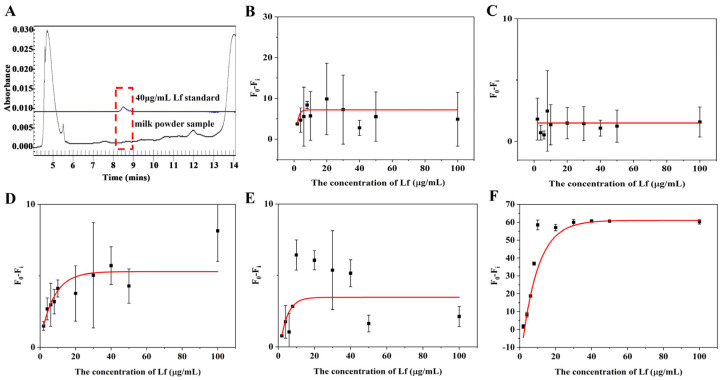
(**A**) Electrophoretogram for Lf content in blank samples by CE. Standard curves for the (**B**) 2, (**C**) 3, (**D**) 5, (**E**) 10, and (**F**) 50 times dilution of matrices obtained by the first milk powder pretreatment method.

**Figure 5:**
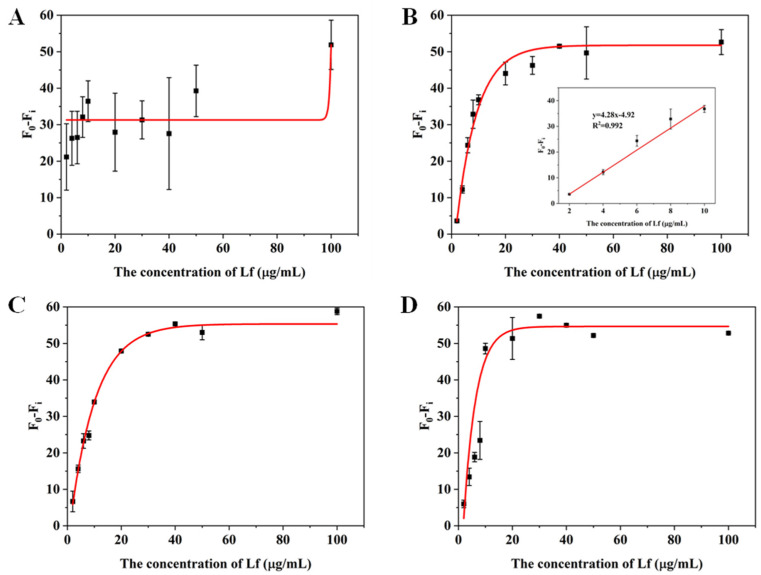
Standard curves for the (**A**) 2, (**B**) 3, (**C**) 5 and (**D**) 10 times dilution of matrices obtained by the second milk powder pretreatment method.

In order to assess the suitability and accuracy of the aptasensor, we performed calibration curves and sensitivity measurements in milk powder matrices. Lf-free infant formula sold in supermarkets was selected as a blank sample and confirmed by the CE approach (Figure 4A). The sample was treated with two reported pre-treatment methods (described in Section 3.5), in which one incorporates a pre-centrifugation step prior to treating the milk powder and adjusts the solution to neutral using NaOH before performing a detection, whereas the other is actually a pre-processing method applied to CE and does not include either of these steps. The matrix solution obtained by these two methods was diluted 2, 3, 5, and 10 times, respectively, with Tris–EDTA buffer. The results are shown in Figure 4 and Figure 5. For the matrix solution obtained by the first treatment method, the matrix interferences are still significant and the error bars are still wide after a 10-fold dilution; in addition, it was later found that there was a need for a 50-fold dilution in order to achieve a good linear relationship.

For the matrix solution obtained by the second treatment method, only a 3-fold dilution was needed to obtain a good linear relationship. The better results of the second method can be attributed to two reasons: the addition of a pre-centrifugation step before the milk powder treatment can more thoroughly remove the fat layer and the precipitate layer of the milk powder, reducing the presence of large molecules in the intermediate clearing layer; in addition, since the isoelectric point of Lf is around 8.0, the use of NaOH to make the assay solution pH-neutral will increase the positive binding of Lf to the aptamer, which may be inhibited in an acidic environment. The standard curve in the inset of Figure 5B that presented a gradual decrease in fluorescence signal as the concentration of Lf increases, was chosen as the matrix standard curve from the point of view of matrix effect and sensitivity. A good linear relationship (R^2^ > 0.99) was obtained from the Lf concentration ranging from 2 to 10 μg/mL and the LOD was calculated to be 0.46 μg/mL (5.55 nM) for the value (F_0_ − F_i_), which also complies with the maximum allowable use such as in the US, EU, and China. With such high sensitivity, the determination of the hidden Lf levels in formula milk could be possible. Even though the slope of the sample matrix was slightly different from that of the standard, it was possible to derive quantitative results for the detection of Lf from the matrix by multiplication of the standard calibration curve with an adjustment coefficient.

### 2.5. Application Evaluation in Real Samples

The recovery experiment was carried out by which three different concentrations of Lf standards were added to the formula milk before pretreatment, and the recoveries were determined based on the matrix calibration curve after treatment with the optimized treatment method. Table 2 indicates that the recoveries of the samples ranged from 89.5 to 104.3% with RSD values less than 8%. The results have supported the robustness and reliability of the aptasensor for the sensitive determination of Lf in the matrix of the formula milk.

The Lf content was determined in three commercially available samples with different Lf concentrations to evaluate the actual application of the aptasensors. For these samples, the CE results were also used as a benchmark. In comparison with the CE results, the three results indicated that the Lf content determined by this aptasensor were roughly in line with the CE values, together with the consistently good average recovery of 94.95–102.68% (Table 3), which indicates that this developed aptasensor has good performance in practical applications. However, the rapid analysis of all samples required only 15 min, significantly shorter than that (approximately 120 min) by the commercial CE methods.

## 3. Experimental Methods

### 3.1. Reagents and Materials

Lactoferrin (Lf, from cow’s milk, purity ≥ 95%) and citric acid were purchased from Shanghai McLean Biochemical Technology Co. (Shanghai, China). The α-lactalbumin (α-La, from cow’s milk, purity ≥ 85%), bovine serum albumin (BSA) (purity ≥ 98%), β-lactoglobulin (β-Lg, from cow’s milk, purity ≥ 90%), lactopontin (LPN, from cow’s milk, purity ≥ 90%) were sourced from Sigma-Aldrich Co., Ltd. (Shanghai, China). All-black 96-well cell culture plates were secured from Shanghai Wolhong Bio-technology Co. (Shanghai, China). The acetic acid (excellent pure), Tris–EDTA buffer (pH 8.0) and PBS buffer (pH 7.2–7.4) were purchased from Beijing Solebo Co., Ltd. (Beijing, China). The NaCl, KCl, MgCl_2_, CaCl_2_, and NaOH were sourced from Sinopharm Chemical Reagent Co., Ltd. (Shanghai, China), and are all analytically pure. The three types of different infant formulas were purchased from regular local supermarkets. The used aptamer was referenced from Qu’s group and its sequence of 5′-TGGTGCTGCCCCTAGTCTCCGGCTGATAGCTGCTTCTTGG-6-FAM-3′ was chemically synthesized and analytically purified by Sangon Biotech (Shanghai, China) Co., Ltd.

Some of the routine equipment used for experiments and sample pre-treatment: circular oscillator (MS3, IKA, Staufen, Germany); high-speed freezing centrifuge (Neofuge 15R, Shanghai, China, Shanghai Lixin Scientific Instrument Co., Ltd.); CAP225D analytical balance with an accuracy of 0.0001 g (Sartorius, Göttingen, Germany). The 0.22 μm cellulose acetate membrane was purchased from Hangzhou Special Paper Industry Co., Ltd. (NEW STAR, Zhejiang, China). The experimental water was ultrapure, and purified by the Milli-Q system (resistivity of 18.2 MΩ-cm@25°C) (Millipore, Bedford, MA, USA). The microplate reader (Multiskan™ FC, Thermo Fisher Scientific, Waltham, MA, USA) was utilized to capture fluorescence changes through the operation at the excitation wavelength (Ex, 485 nm) and the emission wavelength (Em, 528 nm). The 5 mL disposable syringes were purchased from Jiangsu Zhengkang Medical Apparatus Co., Ltd. (Changzhou, China) The 50 µm id-fused silica capillary with an overall length of 32.6 cm was sourced from Sino Sumtech (Handan, Hebei, China), of which the distance between the inlet end and the straight detection window is 20.3 cm. HPCE (Model HPCE512) provided a UV detector with a 214 nm filter supplied by Hanon Group. (Jinan, China).

### 3.2. Optimization of Conditions

Firstly, we optimized the aptamer concentrations at 10, 50, 100, 150, 200, 250, and 300 nM. The Lf aptamer (90 µL) was incubated with an Lf solution (100 µL, 40 µg/mL) for 10 min at room temperature before experimenting. In addition, the reaction time between the aptamer (90 µL, 50 nM) and the Lf (100 µL, 40 µg/mL) was optimized. The aptamer was incubated with Lf at room temperature. Samples in 96-well plates were tested at five-minute intervals for 40 min. In addition, the buffer solutions were optimized. The Lf aptamer (90 µL, 50 nM) was incubated with an Lf solution (100 µL, different concentrations, 0, 30, 60 µg/mL) in different buffers: Tris–EDTA, PBS, H_2_O, citrate buffer, and boric acid buffer for 10 min. The Lf aptamer (90 µL, 50 nM) was incubated with the Lf solution (100 µL, 40 µg/mL) for 10 min at different temperatures: 0, 25, and 37 °C. Finally, the ionic species and ionic strength were optimized. The Lf aptamer (90 µL, 50 nM) and the Lf solution (100 µL, 40 µg/mL) were incubated for 10 min with different ionic species Na^+^, K^+^, Ca^2+^, Mg^2+^, and at different concentrations of 50 mM and 100 mM.

### 3.3. Detection Method

Under the optimal conditions (the concentrations of aptamers, incubation times, the buffer solution, the reaction temperature, the ionic species and the ionic strength), aptamer (90 μL, 50 nM) was incubated with Lf standard (100 μL, 0, 2, 4, 6, 8, 10, 20, 30, 40, 50, and 100 μg/mL) in an all-black 96-well cell plate. Fluorescence values were determined using the microplate reader.

### 3.4. Evaluation of Specificity

To examine the specificity of the aptasensor, four proteins typically present in milk were measured, consisting of α-La, β-Lg, BSA, and LPN, and all set up controls with high concentrations. Various proteins (100 μL, 3, 6, 12, 25, and 50 μg/mL) were incubated with aptamers (90 μL, 50 nM) at room temperature in an all-black 96-well cell plate. After 10 min, fluorescence values were determined using the microplate reader.

### 3.5. Sample Processing and Testing

To evaluate the actual application of the aptasensors, our starting point was a selection of milk powder pre-treatments. The two methods are as follows:

1. Weigh the 500 mg of blank milk powder dissolved in acetic acid solution, vortex mix, so that the concentration reaches 50 mM, centrifuges at 8000 rpm at 4 °C for 10 min to obtain three layers of samples, from top to bottom of the fat layer, the clear liquid layer, the layer of protein precipitation. The intermediate clear liquid layer is aspirated through the 0.22 μm cellulose acetate membrane and stores at 4 °C for spare parts.
2. Weigh the 500 mg of blank milk powder dissolved in water, vortex mix, incubate in a water bath at 40 °C for 30 min, then stand at room temperature for 15 min, centrifuge at 8000 r/min at 4 °C for 15 min, and the supernatant is added with the acetic acid solution, then centrifuges at 4 °C for 10 min at 8000 r/min. The supernatant is added with 1 M NaOH to make it pH neutral, the final concentration is 50 mM, which is filtered through 0.22 μm cellulose acetate membrane and stores at 4 °C.

In the matrix spiking-based linearity experiments, an Lf-free formula was selected as a blank sample, which was treated with an optimized pre-treatment method. The treated matrix solution was used to dilute the Lf standards to concentrations of 0, 2, 4, 6, 8, 10, 20, 30, 40, 50, and 100 μg/mL, and the linearity with the Lf concentration was determined based on the fluorescence reduction values. In the matrix spiking recovery test, the difference was that the Lf standard was added prior to the milk powder treatment, and three Lf concentrations of 4, 8, and 10 μg/mL were selected for the determination of recovery according to the matrix standard curve. In the actual milk powder application, the milk powder was sensed directly after pre-treatment, and the Lf content in the milk powder was determined based on the fluorescence value and matrix calibration curve.

### 3.6. Capillary Electrophoresis Assay

The method for the determination of Lf in milk powder using HPCE was modified from that reported by Li et al. [51]. The fused silica capillary was first activated with 1 M NaOH for 20 min and then closed with water. The electrophoresis buffer solution consisted of 40 mM of NaH_2_PO_4_, 40 mM of H_3_PO_4_, and 5 mM of Brij 35. During the assay, the capillary was rinsed successively with 0.1 M of NaOH, water, and electrophoresis buffer for 3 min between each CE run. Samples were injected at 0.5 psi for 10 s on the desired time. Electrophoretic separation conditions were: high voltage at 15 kV (as inlet to the anode), temperature at 22 °C.

## 4. Conclusions

In conclusion, a self-responsive and user-friendly fluorescence aptasensor was developed for the sensitive detection of Lf in dairy products dependent on the double actions of aptamers functioning both as a target-specific recognition element and as a signaling donor coupled to a fluorescent moiety. The specific binding between the aptamer and Lf through the self-assembly folding of the aptamer induces turn-off fluorescence. Direct recognition and detection eliminate the unnecessary use of toxic dyes and complicated optimization commonly associated with free-label aptasensors, and avoid some of the sophisticated designs that would limit their ease of use. Under the optimized conditions, the proposed aptasensor demonstrates favorable performances including low detection limits, high stability and specificity, as well as a good linear relationship and reliable recoveries in spiked formula milk. In addition, the aptasensor achieved determination of Lf with an accuracy of 89.5–104.3% in three samples of milk formula, and the results were validated by the HPCE method. Thus, the fluorescence aptasensor, through only a “mix and detect” operation, offers significant advantages as a user-friendly and cost-effective detection strategy.

## Figures and Tables

**Table 1:** Performance comparison of various methods for detecting Lf.

Method	Linear Range	LOD	References
Capillary electrophoresis	0.312–9.984 μg/mL	0.078 µg/mL	[22]
Liquid chromatography	10–10^3^ µg/mL	500 µg/mL (liquid samples)	[43]
RP-HPLC	0–5 µg/mL	1 µg/mL	[44]
ESI-MS	10–10^3^ nmol L	0.3 × 10^−2^ mg/g	[45]
Microfluidic paper	0–10^3^ µg/mL	100 µg/mL	[46]
ELISA	0–150 µg/mL	0.018 µg/mL	[47]
Surface plasmon resonance	0.5–3.5 µmol/L	0.28 µmol/L	[48]
Electrochemical sensor	0.1–10 mg/mL	0.1 mg/mL	[49]
Fluorescent aptasensor	20–500 nmol/L	3 nmol/L	[50]
Self-responsive aptasensor	2–10 μg/mL	0.68 μg/mL	This work

**Table 2:** Recovery tests in milk powder matrix.

Added (μg/mL)	Found (μg/mL)	Recovery (%)	RSD (%)
4	4.17 ± 0.553	104.3 ± 13.82	7.66
8	7.16 ± 0.111	89.5 ± 1.39	0.9
10	9.00 ± 0.301	90.0 ± 3.01	1.93

**Table 3:** Comparison of the test results and methods for three samples of powdered milk (*n* = 3).

Sample	Found (μg/mL)	HPCE (μg/mL)	Consistency (%)
1	0	0	100%
2	16.73	17.62	94.95
3	23.78	23.16	102.68

## Data Availability

The data presented in this study are available in the article itself.
